# Peroxisomal ABC transporters: functions and mechanism

**DOI:** 10.1042/BST20150127

**Published:** 2015-10-09

**Authors:** Alison Baker, David J. Carrier, Theresia Schaedler, Hans R. Waterham, Carlo W. van Roermund, Frederica L. Theodoulou

**Affiliations:** * Centre for Plant Sciences, School of Molecular and Cellular Biology, University of Leeds, Leeds LS2 9JT, U.K.; † Biological Chemistry and Crop Protection Department, Rothamsted Research, Harpenden, AL5 2JQ, U.K.; ‡ Laboratory of Genetic Metabolic Diseases, Departments of Pediatrics and Clinical Chemistry, Academic Medical Center, University of Amsterdam, 1105 AZ Amsterdam, The Netherlands.

**Keywords:** ATP-binding cassette (ABC) transporter, acyl-CoA, asymmetry, β-oxidation, peroxisome, thioesterase

## Abstract

Peroxisomes are arguably the most biochemically versatile of all eukaryotic organelles. Their metabolic functions vary between different organisms, between different tissue types of the same organism and even between different developmental stages or in response to changed environmental conditions. New functions for peroxisomes are still being discovered and their importance is underscored by the severe phenotypes that can arise as a result of peroxisome dysfunction. The β-oxidation pathway is central to peroxisomal metabolism, but the substrates processed are very diverse, reflecting the diversity of peroxisomes across species. Substrates for β-oxidation enter peroxisomes via ATP-binding cassette (ABC) transporters of subfamily D; (ABCD) and are activated by specific acyl CoA synthetases for further metabolism. Humans have three peroxisomal ABCD family members, which are half transporters that homodimerize and have distinct but partially overlapping substrate specificity; *Saccharomyces cerevisiae* has two half transporters that heterodimerize and plants have a single peroxisomal ABC transporter that is a fused heterodimer and which appears to be the single entry point into peroxisomes for a very wide variety of β-oxidation substrates. Our studies suggest that the *Arabidopsis* peroxisomal ABC transporter AtABCD1 accepts acyl CoA substrates, cleaves them before or during transport followed by reactivation by peroxisomal synthetases. We propose that this is a general mechanism to provide specificity to this class of transporters and by which amphipathic compounds are moved across peroxisome membranes.

## Introduction

### Peroxisome functions and the requirement for transport

Peroxisomes are single membrane-delimited organelles found in almost all eukaryotic cells. Originally named for their peroxidative metabolism, it has now become appreciated that they possess multiple, diverse metabolic capabilities and that these vary between organisms and even between cell types or developmental stages of the same organism [1,2] New functions for peroxisomes are still being discovered [3–5]. One conserved function of peroxisomes is β-oxidation, a pathway by which acyl groups are degraded two carbons at a time following activation to the corresponding acyl CoA thioester by a specific acyl CoA synthetase. Many different substrates can be processed by peroxisomal β-oxidation. The degradation of fatty acids by β-oxidation occurs exclusively in the peroxisome in fungi and plants whereas, in mammals, peroxisomes handle degradation of branched, very long chain and dicarboxylic acids which are exported to mitochondria as C-16 units for completion of catabolism [1,2]. Peroxisomal β-oxidation is also important for synthesis of some bioactive molecules such as docosahexaenoic acid in mammals [2] and the plant hormone jasmonic acid (JA) which is synthesized from its precursor 12-oxophytodienoic acid (OPDA) by three rounds of β-oxidation [6].

The metabolic activities of peroxisomes require that many substrates, products and cofactors have to cross the peroxisomal membrane to and from the cytosol. A channel-forming protein, PXMP2 (peroxisomal membrane protein 2), which is present in the peroxisomal membrane, has been shown *in vitro* to allow transmembrane passage of small solute molecules with a molecular mass below 400 Da [7,8]. Based on this finding, it has been postulated that small molecules below 400 Da can cross the peroxisomal membrane passively via this protein, whereas others suggest a regulated process [9]. Similarly, plant peroxisome membranes possess a pore-forming activity [10]. For bulky solute molecules (>400 Da); however, the peroxisomal membrane is an impermeable barrier and transporter proteins are required [7].

Relatively few peroxisomal transporters have been identified but it is well-established that peroxisome localized members of ATP-binding cassette (ABC) subfamily D mediate uptake of substrates for β-oxidation [10]. The ABCD family also includes the human endoplasmic reticulum (ER)/lysosome localized PMP69 (69kDa peroxisome membrane protein)/ABCD4 which is implicated in cobalamin transport [11] and an *Arabidopsis* protein AtABCD2 which is in the chloroplast but for which there is little functional information [12]. However, this review will focus on the peroxisomal members of this family.

## Yeast ABCD transporters

In *S. cerevisiae*, two half transporters, termed Pxa1p (peroxisomal ABC transporter1) and Pxa2p (peroxisomal ABC transporter2) are involved in long chain fatty acyl-CoA transport across the peroxisomal membrane [13–16]. Single knockouts *pxa1∆* and *pxa2∆* cannot utilize oleate (C18:1) as a sole carbon source and display reduced β-oxidation [13,14]. The double mutant *pxa1/pxa2∆* does not have an enhanced phenotype in comparison with the single mutants and protein–protein interaction studies demonstrated that the two half size transporters heterodimerize to form a fully functional transporter [15], with the central domain of the C-terminal region in Pxa2p being required for the dimerization process [17].

Non-esterified fatty acids can enter the peroxisome independently of the ABC transporter and are activated by the peroxisomal acyl-CoA synthetase, Faa2p (fatty acid activation protein 2), prior to β-oxidation [14], a process which requires the activity of the peroxisomal ATP carrier, Ant1p (adenine nucleotide transporter1) [18,19]. Interestingly, a peroxisomal ABC transporter homologous to ScPxa1/2p is not essential for the growth of the oleaginous yeast, *Yarrowia lipolytica* on fatty acids, but growth is impaired when YlPxa1/2p and YlAnt1p are deleted [20].

## Plant ABCD transporters

**Figure 1:**
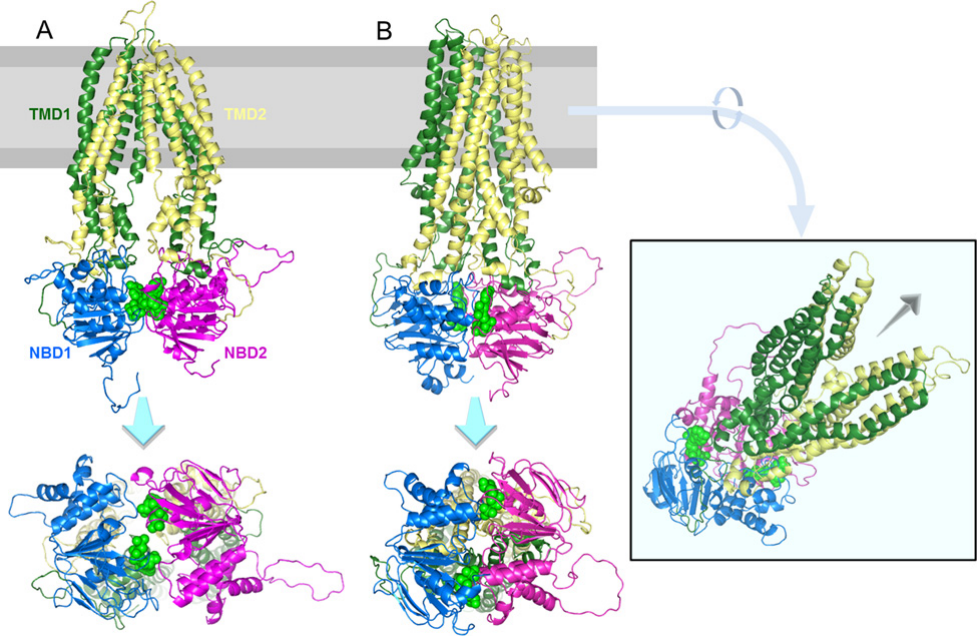
Molecular models showing putative inward and outward conformations of *Arabidopsis* CTS Models of CTS are based on the structures of (**A**) ABCB10 [52], with bound AMP–PCP (β,γ-methylene adenosine 5′-triphosphate) (green spheres) in an inward-facing conformation [2YL4.pdb replaced in database by 4AYT.pdb; the two structures can be superimposed with a RMSD of 0.19 Å (1 Å=0.1 nm)] and (**B**) Sav1866 [53] open-outward ADP-bound complex in which the two NBDs (blue and purple) are closely packed with two nucleotides (green spheres), sandwiched between them (2HYD.pdb). The NBDs face the cytosol [39], views are from side-on (top image) and bottom-up (bottom image) and an angled view to show the pore, the possible site of substrate release, created in the open conformation. Note the domain swapping where TMD2 (yellow) contacts NBD1 (blue) and TMD1 (green) contacts NBD2 (purple).

*Arabidopsis* AtABCD1 is the most studied and best understood plant peroxisomal ABC transporter and was identified and named independently by at least four different groups [CTS (COMATOSE), PXA1 (peroxisomal ABC transporter1), PED3 (peroxisome defective 3) and ACN2 (acetate non utilizing 2)] [21–24]. The *CTS* gene is expressed throughout the plant and encodes a full-sized transporter with two homologous but distinct halves fused in a heterodimer in the arrangement [TMD1] (the transmembrane domain)–[NBD1] (nucleotide-binding domain)–[TMD2]–[NBD2] (Figure 1). *cts* Mutants are defective in lipid mobilization, require an exogenous carbon source for seedling establishment and accumulate acyl-CoAs [22], suggesting that CTS mediates the transport of fatty acyl-CoAs into the peroxisome.

CTS has also been implicated in the import of a wide range of other substrates: the JA precursor, OPDA [6,25], acetate [24], the auxin precursors 2,4-dichlorophenoxybutyric acid and indole butyric acid (IBA) [21,23], precursors of ubiquinone synthesis [3] and cinnamic acid which is required for the synthesis of benzoic acid [4]. In *Arabidopsis*, the *CTS* (*COMATOSE*) locus was identified in genetic screens for positive regulators of germination [22]. Subsequent studies demonstrated that OPDA accumulation underlies the germination defect through up-regulation of a transcription factor, *ABI5* (*ABA insensitive 5*) which inhibits seed coat rupture [25,26]. Although the presumptive substrates for CTS appear superficially dissimilar in size and shape, they all share a common feature in having an acyl chain of at least four carbons terminating in a carboxy group that is potentially esterified to a CoA. Taken together, these data indicate that CTS acts as a gateway into the peroxisome for β-oxidation, influencing a wide range of metabolic and signalling processes.

In contrast with dicotyledonous plants, cereals contain two *ABCD1* homologues arising from a gene duplication that occurred prior to the divergence of the Gramineae [27]. Functional studies in barley (*Hordeum vulgare*) suggest these two genes are in the process of undergoing neofunctionalization. Whereas both *HvABCD1* and *2* are involved in fatty acid and IBA metabolism, only *HvABCD2* could complement the *Arabidopsis cts* germination phenotype, pointing to a role for the latter in OPDA metabolism [28]. ABCD1 proteins are also implicated in control of seed size in *Arabidopsis*, barley and tomato, since mutants in all these species have reduced seed weight, but the biochemical basis for this is currently unclear [28,29].

## Human ABCD transporters

In mammalian cells, peroxisomes are involved in a number of important metabolic pathways, including the α- and β-oxidation of fatty acids and the biosynthesis of ether phospholipids and bile acids [2]. Mammalian peroxisomes contain three members of the ABC family: ABCD1 [adrenoleukodystrophy protein (ALDP); 30], ABCD2 [adrenoleukodystrophy-related protein (ALDR); 31] and ABCD3 [70 kDa peroxisomal membrane protein (PMP70); 32]. All three proteins are half-ABC transporters and need to dimerize to constitute a full, active transporter.

Expression of the human (Hs) peroxisomal ABC transporters in yeast followed by functional characteriszation has shown that the ABCD transporters have distinct but overlapping specificities for different acyl-CoA esters. Most hydrophobic C24:0-CoA and C26:0-CoA esters are preferentially transported by HsABCD1, whereas C22:0-CoA, C22:6-CoA and C24:6-CoA are preferentially transported by HsABCD2. Substrates such as long-chain unsaturated acyl-CoAs, 2-methyl branched-chain acyl-CoAs including pristanoyl-CoA and long-chain dicarboxylic CoA esters are preferentially transported by HsABCD3. Although an active heterodimer of ABCD1 and ABCD2 has been reported [33], these yeast studies also demonstrated that HsABCD1, HsABCD2 and HsABCD3 can function as active homodimers [34].

The overlapping activities of the peroxisomal ABC transporters are also evident from the residual C26:0-CoA β-oxidation activity that is found in fibroblasts of patients with X-linked adrenoleucodystrophy (X-ALD), the most common peroxisomal disorder caused by mutations in the *ABCD1* gene [35]. This *ABCD1* defect causes impaired peroxisomal β-oxidation of very long-chain fatty acids (VLCFAs, ≥C22:0) and consequently, accumulation of VLCFAs especially in brain and adrenal glands. The residual C26:0-CoA β-oxidation activity is mediated by ABCD3. In addition, the VLCFA β-oxidation activity in fibroblasts from X-ALD patients can be rescued by overexpression of *ABCD2* or *ABCD3* [35]. Ferdinandusse et al. [5] recently described a new peroxisomal disorder caused by mutations in the *ABCD3* gene. A patient with hepatosplenomegaly and severe liver disease had a striking accumulation of bile acid intermediates DHCA (dihydroxycholestanoic acid) and THCA, indicating a role of HsABCD3 in the transport of these compounds. The comparatively high expression level of *ABCD3* in organs such as liver, heart, kidney, muscle and brain [35], suggests that HsABCD3 is also (indirectly) involved in fatty acid oxidation as an energy generating process [34]. In line with this, it was shown that overexpression or silencing of *ABCD3* in fibroblasts alters palmitate β-oxidation activity accordingly [35,36].

## Mechanism of fatty acid transport

Both human and plant ABCD genes can partially rescue the β-oxidation and growth phenotypes of the *pxa1/pxa2∆* double mutant, reflecting aspects of conserved function with respect to fatty acid metabolism [28,37–39]. However, human ABCD1 cannot complement the *cts* plant mutant phenotype and human ABCD2 can only complement the germination defect supporting the notion that the plant transporters have a broader substrate specificity [40]. The precise nature of the molecular species recognized by the transporters and the mechanism of transport have been controversial. Accumulating evidence supports the notion that ABCD proteins transport the CoA esters of fatty acids across the peroxisomal membrane [13,22,34,37–39,41]. Owing to their fragile nature, transport experiments with isolated peroxisomes are challenging; however, RNAi mediated suppression of *Trypansoma brucei* ABCD transporter Gat1 (glycosomal ABC transporter1) reduced incorporation of ^14^C-C18:1-CoA into purified glycosomes [41] and isolated human fibroblast peroxisomes could oxidize C26:0-CoA [42]. Two models have been described for acyl-CoA transport: in the first model, esterified fatty acids are delivered directly to the peroxisomal matrix [42], whereas in the other model acyl-CoA is hydrolysed during transport and re-esterified in the peroxisomal lumen by an acyl-CoA synthetase [43–45].

**Figure 2:**
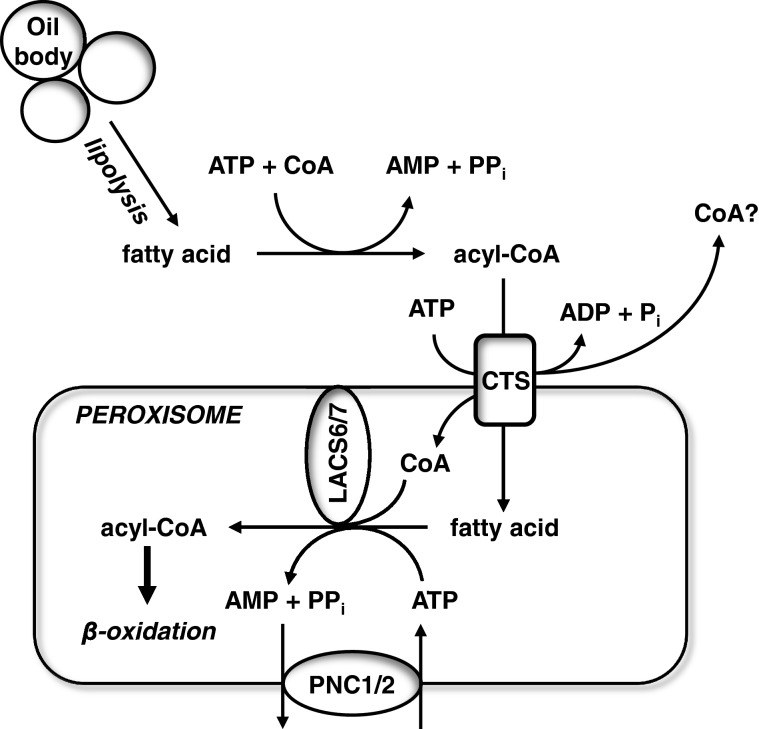
Proposed transport mechanism for CTS Fatty acids are released from oil body stores of triacylglycerol by lipolysis. Cytosolic or microsomal acyl CoA synthetases activate non-esterified fatty acids to the corresponding acyl-CoA esters. CTS accepts acyl-CoAs; once bound, the intrinsic thioesterase activity of the transporter releases fatty acids which may be flip-flopped in the membrane and released into the peroxisome, where they are re-esterified by the activity of LACS6 and 7. A pool of LACS6/7 protein is physically associated with CTS on the lumenal side of the membrane [45] and ATP for the activation reaction is provided by peroxisomal adenine nucleotide carriers PNC1/2. The CoA moiety is thought to be imported into the peroxisome via the ABC transporter or alternatively may be released into the cytosol.

Interestingly, HsABCD1 and HsABCD2 could only complement *pxa1/pxa2∆* for β-oxidation of C22:0 and C24:0 if the peroxisomal long chain fatty acid peroxisomal synthase Faa2p was present [43]. When expressed in yeast, *Arabidopsis* CTS showed ATPase activity that could be stimulated by fatty acid acyl CoA (FA-CoA) derivatives and the ability to complement the yeast *pxa1pxa2Δ* mutant for growth on oleate as a sole carbon source [27,39]. CTS was also dependent upon the presence in the peroxisome of Faa2p or the equivalent Arabidopsis long chain acyl CoA synthetases (LACS)6 and/or LACS7 for functional complementation of the *pxa1/pxa2/faa2∆* mutant [45], which is consistent with the *in planta* requirement for LACS6 and 7 for fatty acid breakdown during *Arabidopsis* seedling establishment [44]. Overall, the requirement for a peroxisomal activation step suggested that the CoA moiety is cleaved off during the transport cycle (Figure 2), which was confirmed in experiments using isotopic labelling of yeast cells with ^18^O, although this study could not reveal the origin of the thioesterase activity [43]. Evidence that it is the ABCD transporter itself came from experiments with membranes from insect cells expressing CTS. These showed ATP stimulated thioesterase activity that was much decreased in membranes from cells expressing a non-functional mutant. This is strongly suggestive of a thioesterase activity intrinsic to the ABC transporter, although there is no domain with homology to either α/β hydrolases or hot-dog fold thioesterases [45]. Whereas a soluble *Arabidopsis* α/β hydrolase, CGI-58 has been proposed to stimulate the activity of CTS *in planta*, *cgi-58* mutants do not exhibit defects in germination or seed oil mobilization [46].

At first sight, cleavage and reactivation of the acyl CoA appears energetically wasteful, since the equivalent of two molecules of ATP are used per activation reaction. However such a mechanism could provide a solution to the problem of how diverse substrates are accepted if the CoA moiety is an important determinant for recognition and cleavage potentially enables separate permeation pathways for the polar (CoA) and hydrophobic (fatty acid) moieties of β-oxidation substrates. Where CoA is released and whether and if so how it enters peroxisomes remains to be determined. At 750 kDa, it is too large to utilize the general pore; so, some means of transport must be necessary to maintain the supply of CoA for β-oxidation. Furthermore, different substrates require different intraperoxisomal synthetases for activation, potentially lending further specificity. Nevertheless why mammals have three ABCD proteins with distinct but overlapping specificity whereas most plants and yeast have a single one with apparently broad substrate specificity is unclear.

## Symmetry and asymmetry in ABCD transporters

**Figure 3:**
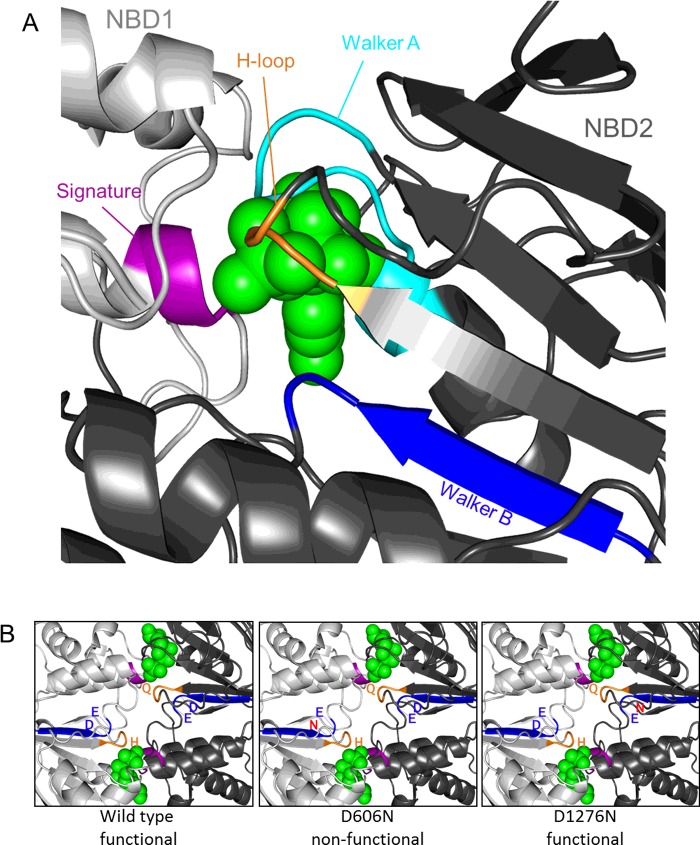
Molecular model showing asymmetry in the two nucleotide binding sites of CTS (**A**) Nucleotide binding between the Walker A (cyan), Walker B (blue) and H-loops (orange) motifs of NBD2 (dark grey) and the signature motif (purple) of NBD1 (light grey). (**B**) In the wild-type protein, the upper site is formed from the signature motif of NBD1 and the Walker motifs and H loop of NBD2 and the lower site is formed from the Walker motifs and H loop of NBD1 and the signature motif of NBD2. In the upper site, the conserved H of the H loop is replaced by Q leading to a degenerate site whereas the lower site has all the consensus amino acids. Mutation of the Walker B aspartate (D606N) in NBD1 leads to two degenerate sites and loss of function whereas the equivalent mutation in NBD2 (D1276N) still retains one consensus site and is functional [48].

A curious feature of the ABCD family is that it contains both homodimeric and heterodimeric transporters which mediate similar transport functions and apparently share the same unusual mechanism. This has implications not only for ATP binding and hydrolysis but also for binding and translocation of the transported substrates. It is well established that the NBDs of ABC transporters form a sandwich dimer with two composite ATP-binding sites comprising the Walker A, Walker B and H-loops of one NBD and the signature motif of the other [47]; Figures 1 and 3. By definition, these sites are identical in homodimeric transporters such as ALDP but they differ in heterodimeric peroxisomal transporters found in yeast and plants. Based on sequence comparisons, Pxa1/2p and CTS have one consensus and one degenerate site and analysis of an allelic series of *cts* mutants demonstrated that the NBDs of CTS are not functionally equivalent (Figure 3B) [48]. Asymmetry appears to have evolved several times independently in the ABC protein superfamily, although the functional consequences of substitutions in conserved motifs have not been investigated in all cases [49]. In CFTR (cystic fibrosis transconductance regulator) and multidrug resistance protein1 (MRP1), nucleotide hydrolysis is rapid at the consensus site whereas nucleotide is bound but turned over slowly at the degenerate site [50,51]. Numerous mutagenesis studies have shown that an efficient ATP hydrolysis cycle at a single consensus site is sufficient to provide all the necessary conformational changes for substrate transport [49].

CTS and Pxa1/2p also exhibit sequence asymmetry in TMDs (Figure 1) which potentially influences binding and translocation of substrates. This may underpin the broader substrate specificity of the heterodimeric transporters although the substrate-binding sites of ABCDs remain to be identified. Intriguingly, both homodimeric and heterodimeric ABCD transporters appear to share a thioesterase-dependent transport mechanism [43,45], so it will be interesting to determine whether there is one or two thioesterase sites in symmetrical transporters such as ALDP.

## Concluding remarks

In recent years, much progress has been made in understanding the physiological functions and biochemical roles of peroxisomal ABC transporters, with the emergence of exciting, novel mechanistic insights. Future challenges include elucidating the transport mechanism in further detail and relating this to the as yet elusive structure of ABC subfamily D proteins.

## Abbreviations

ABC: ATP-binding cassette
ABCD: ABC transporter of subfamily D
ABI5: ABA insensitive 5
ACN2: acetate non utilizing 2
ALDP: adrenoleucodystrophy protein
AMPPCP: β,γ-methyleneadenosine 5′-triphosphate
Ant1p: adenine nucleotide transporter1
CFTR: cystic fibrosis transconductance regulator
CTS: comatose
Faa2p: fatty acid activation protein 2
FA-CoA: fatty acid acyl CoA
Gat1: glycosomal ABC transporter1
IBA: indole butyric acid
JA: jasmonic acid
LACS: long chain acyl-CoA synthetases
MRP1: multidrug resistance protein1
NBD: nt-binding domain
OPDA: 12-oxophytodienoic acid
PED3: peroxisome defective 3
PMP69: 69kDa peroxisome membrane protein
PNC: peroxisome nucleotide carrier
PXA1: peroxisomal ABC transporter1
PXMP2: peroxisomal membrane protein 2
TMD: transmembrane domain
VLCFA: very long-chain fatty acid
X-ALD: X-linked adrenoleucodystrophy

## References

[1] Hu J., Baker A., Bartel B., Linka N., Mullen R.T., Reumann S., Zolman B.K. (2012). Plant peroxisomes: biogenesis and function. Plant Cell.

[2] Wanders R.J.A., Waterham H.R. (2006). Biochemistry of mammalian peroxisomes revisited. Annu. Rev. Biochem..

[3] Block A., Widhalm J.R., Fatihi A., Cahoon R.E., Wamboldt Y., Elowsky C., Mackenzie S.A., Cahoon E.B., Chapple C., Dudareva N., Basset G.J. (2014). The origin and biosynthesis of the benzenoid moiety of ubiquinone (coenzyme Q) in *Arabidopsis*. Plant Cell.

[4] Bussell J.D., Reichelt M., Wiszniewski A.A.G., Gershenzon J., Smith S.M. (2014). Peroxisomal ATP-binding cassette transporter COMATOSE and the multifunctional protein abnormal inflorescence meristem are required for the production of benzoylated metabolites in arabidopsis seeds. Plant Physiol..

[5] Ferdinandusse S., Jimenez-Sanchez G., Koster J., Denis S., Van Roermund C.W., Silva-Zolezzi I., Moser A.B., Visser W.F., Gulluoglu M., Durmaz O. (2015). A novel bile acid biosynthesis defect due to a deficiency of peroxisomal ABCD3. Hum. Mol. Genet..

[6] Theodoulou F.L., Job K., Slocombe S.P., Footitt S., Holdsworth M., Baker A., Larson T.R., Graham I.A. (2005). Jasmonic acid levels are reduced in comatose ATP-binding cassette transporter mutants. Implications for transport of jasmonate precursors into peroxisomes. Plant Physiol..

[7] Antonenkov V.D., Hiltunen J.K. (2006). Peroxisomal membrane permeability and solute transfer. Biochim. Biophys. Acta.

[8] Rokka A., Antonenkov V.D., Soininen R., Immonen H.L., Pirila P.L., Bergmann U., Sormunen R.T., Weckstram M., Benz R., Hiltunen J.K. (2009). Pxmp2 is a channel-forming protein in mammalian peroxisomal membrane. PLoS One.

[9] Kunze M., Hartig A. (2013). Permeability of the peroxisomal membrane: lessons from the glyoxylate cycle. Front. Physiol..

[10] Linka N., Theodoulou F.L. (2013). Metabolite transporters of the plant peroxisomal membrane: known and unknown. Subcell. Biochem..

[11] Coelho D., Kim J.C., Miousse I.R., Fung S., du Moulin M., Buers I., Suormala T., Burda P., Frapolli M., Stucki M. (2012). Mutations in ABCD4 cause a new inborn error of vitamin B-12 metabolism. Nat. Genet..

[12] Hurlock A.K., Roston R.L., Wang K., Benning C. (2014). Lipid trafficking in plant cells. Traffic.

[13] Verleur N., Hettema E.H., VanRoermund C.T., Tabak H.F., Wanders R.A. (1997). Transport of activated fatty acids by the peroxisomal ATP- binding-cassette transporter Pxa2 in a semi-intact yeast cell system. Eur. J. Biochem..

[14] Hettema E.H., van Roermund C.W., Distel B., van den Berg M., Vilela C., Rodrigues-Pousada C., Wanders R.J., Tabak H.F. (1996). The ABC transporter proteins Pat1 and Pat2 are required for import of long-chain fatty acids into peroxisomes of *Saccharomyces cerevisiae*. EMBO J..

[15] Shani N., Valle D. (1996). A Saccharomyces cerevisiae homolog of the human adrenoleukodystrophy transporter is a heterodimer of two half ATP-binding cassette transporters. Proc. Natl. Acad. Sci. U.S.A..

[16] Swartzman E.E., Viswanathan M.N., Thorner J. (1996). The PAL1 gene product is a peroxisomal ATP-binding cassette transporter in the yeast *Saccharomyces cerevisiae*. J. Cell Biol..

[17] Chuang C.Y., Chen L.Y., Fu R.H., Chen S.M., Ho M.H., Huang J.M., Hsu C.C., Wang C.C., Chen M.S., Tsai R.T. (2014). Involvement of the carboxyl-terminal region of the yeast peroxisomal half ABC transporter Pxa2p in its interaction with Pxa1p and in transporter function. PLoS One.

[18] van Roermund C.W.T., Drissen R., van den Berg M., Ijlst L., Hettema E.H., Tabak H.F., Waterham H.R., Wanders R.J.A. (2001). Identification of a peroxisomal ATP carrier required for medium-chain fatty acid beta-oxidation and normal peroxisome proliferation in *Saccharomyces cerevisiae*. Mol. Cell. Biol..

[19] Palmieri L., Rottensteiner H., Girzalsky W., Scarcia P., Palmieri F., Erdmann R. (2001). Identification and functional reconstitution of the yeast peroxisomal adenine nucleotide transporter. EMBO J..

[20] Dulermo R., Gamboa-Melendez H., Ledesma-Amaro R., Thevenieau F., Nicaud J.M. (2015). Unraveling fatty acid transport and activation mechanisms in *Yarrowia lipolytica*. Biochim. Biophys. Acta.

[21] Hayashi M., Nito K., Takei-Hoshi R., Yagi M., Kondo M., Suenaga A., Yamaya T., Nishimura M. (2002). Ped3p is a peroxisomal ATP-binding cassette transporter that might supply substrates for fatty acid beta-oxidation. Plant Cell Physiol..

[22] Footitt S., Slocombe S.P., Larner V., Kurup S., Wu Y.S., Larson T., Graham I., Baker A., Holdsworth M. (2002). Control of germination and lipid mobilization by COMATOSE, the *Arabidopsis* homologue of human ALDP. EMBO J..

[23] Zolman B.K., Silva I.D., Bartel B. (2001). The *Arabidopsis* pxa1 mutant is defective in an ATP-binding cassette transporter-like protein required for peroxisomal fatty acid beta-oxidation. Plant Physiol..

[24] Hooks M.A., Turner J.E., Murphy E.C., Johnston K.A., Burr S., Jaroslawski S. (2007). The *Arabidopsis* ALDP protein homologue COMATOSE is instrumental in peroxisomal acetate metabolism. Biochem. J..

[25] Dave A., Hernandez M.L., He Z., Andriotis V.M.E., Vaistij F.E., Larson T.R., Graham I.A. (2011). 12-Oxo-phytodienoic acid accumulation during seed development represses seed germination in arabidopsis. Plant Cell.

[26] Kanai M., Nishimura M., Hayashi M. (2010). A peroxisomal ABC transporter promotes seed germination by inducing pectin degradation under the control of ABI5. Plant J..

[27] Nyathi Y., Zhang X., Baldwin J.M., Bernhardt K., Johnson B., Baldwin S.A., Theodoulou F.L., Baker A. (2012). Pseudo half-molecules of the ABC transporter, COMATOSE, bind Pex19 and target to peroxisomes independently but are both required for activity. FEBS Lett..

[28] Mendiondo G.M., Medhurst A., van Roermund C.W., Zhang X., Devonshire J., Scholefield D., Fernández J., Axcell B., Ramsay L., Waterham H.R. (2014). Barley has two peroxisomal ABC transporters with multiple functions in β-oxidation. J. Exp. Bot..

[29] Orsi C.H., Tanksley S.D. (2009). Natural variation in an ABC transporter gene associated with seed size evolution in tomato species. PLos Genet.

[30] Mosser J., Douar A.M., Sarde C.O., Kioschis P., Feil R., Moser H., Poustka A.M., Mandel J.L., Aubourg P. (1993). Putative X-linked adrenoleukodystrophy gene shares unexpected homology with ABC transporters. Nature.

[31] Lombard-Platet G., Savary S., Sarde C.O., Mandel J.L., Chimini G. (1996). A close relative of the adrenoleukodystrophy (ALD) gene codes for a peroxisomal protein with a specific expression pattern. Proc. Natl. Acad. Sci. U.S.A..

[32] Kamijo K., Taketani S., Yokota S., Osumi T., Hashimoto T. (1990). The 70-kDa peroxisomal membrane protein is a member of the Mdr (P-glycoprotein)-related ATP-binding protein superfamily. J. Biol. Chem..

[33] Geillon F., Gondcaille C., Charbonnier S., Van Roermund C.W., Lopez T.E., Dias A.M.M., Pais de Barros J.-P., Arnould C., Wanders R.J., Trompier D., Savary S. (2014). Structure-function analysis of peroxisomal ATP-binding cassette transporters using chimeric dimers. J. Biol. Chem..

[34] van Roermund C.W.T., Ijlst L., Wagemans T., Wanders R.J.A., Waterham H.R. (2014). A role for the human peroxisomal half-transporter ABCD3 in the oxidation of dicarboxylic acids. Biochim. Biophys. Acta.

[35] Kemp S., Theodoulou F.L., Wanders R.J.A. (2011). Mammalian peroxisomal ABC transporters: from endogenous substrates to pathology and clinical significance. Br. J. Pharmacol..

[36] Di Benedetto R., Denti M.A., Salvati S., Sanchez M., Attorri L., David G., Di Biase A. (2008). RNAi-mediated silencing of ABCD3 gene expression in rat C6 glial cells: a model system to study PMP70 function. Neurochem. Int..

[37] van Roermund C.W., Visser W.F., Ijlst L., van Cruchten A., Boek M., Kulik W., Waterham H.R., Wanders R.J. (2008). The human peroxisomal ABC half transporter ALDP functions as a homodimer and accepts acyl-CoA esters. FASEB J..

[38] van Roermund C.W., Visser W.F., Ijlst L., Waterham H.R., Wanders R.J. (2011). Differential substrate specificities of human ABCD1 and ABCD2 in peroxisomal fatty acid beta-oxidation. Biochim. Biophys. Acta.

[39] Nyathi Y., De Marcos Lousa C., van Roermund C.W., Wanders R.J., Johnson B., Baldwin S.A., Theodoulou F.L., Baker A. (2010). The *Arabidopsis* peroxisomal ABC transporter, comatose, complements the *Saccharomyces cerevisiae* pxa1 pxa2Delta mutant for metabolism of long-chain fatty acids and exhibits fatty acyl-CoA-stimulated ATPase activity. J. Biol. Chem..

[40] Zhang X., De Marcos Lousa C., Schutte-Lensink N., Ofman R., Wanders R.J., Baldwin S.A., Baker A., Kemp S., Theodoulou F.L. (2011). Conservation of targeting but divergence in function and quality control of peroxisomal ABC transporters: an analysis using cross-kingdom expression. Biochem. J..

[41] Igoillo-Esteve M., Mazet M., Deumer G., Wallemacq P., Michels P.A. (2011). Glycosomal ABC transporters of Trypanosoma brucei: characterisation of their expression, topology and substrate specificity. Int. J. Parasitol..

[42] Wiesinger C., Kunze M., Regelsberger G., Forss-Petter S., Berger J. (2013). Impaired very long-chain acyl-coA β-oxidation in human X-linked adrenoleukodystrophy fibroblasts is a direct consequence of ABCD1 transporter dysfunction. J. Biol. Chem..

[43] van Roermund C.W., Ijlst L., Majczak W., Waterham H.R., Folkerts H., Wanders R.J., Hellingwerf K.J. (2012). Peroxisomal fatty acid uptake mechanism in *Saccharomyces cerevisiae*. J. Biol. Chem..

[44] Fulda M., Schnurr J., Abbadi A., Heinz E., Browse J. (2004). Peroxisomal Acyl-CoA synthetase activity is essential for seedling development in *Arabidopsis thaliana*. Plant Cell.

[45] De Marcos Lousa C., van Roermund C.W., Postis V.L., Dietrich D., Kerr I.D., Wanders R.J., Baldwin S.A., Baker A., Theodoulou F.L. (2013). Intrinsic acyl-CoA thioesterase activity of a peroxisomal ATP binding cassette transporter is required for transport and metabolism of fatty acids. Proc. Natl. Acad. Sci. U.S.A..

[46] Park S., Gidda S.K., James C.N., Horn P.J., Khuu N., Seay D.C., Keereetaweep J., Chapman K.D., Mullen R.T., Dyer J.M. (2013). The α/β hydrolase CGI-58 and peroxisomal transport protein PXA1 coregulate lipid homeostasis and signaling in arabidopsis. Plant Cell.

[47] Smith P.C., Karpowich N., Millen L., Moody J.E., Rosen J., Thomas P.J., Hunt J.F. (2002). ATP Binding to the motor domain from an ABC transporter drives formation of a nucleotide sandwich dimer. Mol. Cell.

[48] Dietrich D., Schmuths H., Lousa C.D.M., Baldwin J.M., Baldwin S.A., Baker A., Theodoulou F.L., Holdsworth M.J. (2009). Mutations in the arabidopsis peroxisomal ABC Transporter COMATOSE allow differentiation between multiple functions in planta: insights from an allelic series. Mol. Biol. Cell.

[49] Procko E., O'Mara M.L., Bennett W.F.D., Tieleman D.P., Gaudet R. (2009). The mechanism of ABC transporters: general lessons from structural and functional studies of an antigenic peptide transporter. FASEB J..

[50] Hou Y.-x., Cui L., Riordan J.R., Chang X.-B. (2000). Allosteric interactions between the two non-equivalent nucleotide binding domains of multidrug resistance protein MRP1. J. Biol. Chem..

[51] Aleksandrov L., Aleksandrov A.A., Chang X.-B., Riordan J.R. (2002). The first nucleotide binding domain of cystic fibrosis transmembrane conductance regulator is a site of stable nucleotide interaction, whereas the second is a site of rapid turnover. J. Biol. Chem..

[52] Shintre C.A., Pike A.C.W., Li Q., Kim J.-I., Barr A.J., Goubin S., Shrestha L., Yang J., Berridge G., Ross J. (2013). Structures of ABCB10, a human ATP-binding cassette transporter in apo- and nucleotide-bound states. Proc. Natl. Acad. Sci. U.S.A..

[53] Dawson R.J.P., Locher K.P. (2006). Structure of a bacterial multidrug ABC transporter. Nature.

